# Pubertal development and prostate cancer risk: Mendelian randomization study in a population-based cohort

**DOI:** 10.1186/s12916-016-0602-x

**Published:** 2016-04-04

**Authors:** Carolina Bonilla, Sarah J. Lewis, Richard M. Martin, Jenny L. Donovan, Freddie C. Hamdy, David E. Neal, Rosalind Eeles, Doug Easton, Zsofia Kote-Jarai, Ali Amin Al Olama, Sara Benlloch, Kenneth Muir, Graham G. Giles, Fredrik Wiklund, Henrik Gronberg, Christopher A. Haiman, Johanna Schleutker, Børge G. Nordestgaard, Ruth C. Travis, Nora Pashayan, Kay-Tee Khaw, Janet L. Stanford, William J. Blot, Stephen Thibodeau, Christiane Maier, Adam S. Kibel, Cezary Cybulski, Lisa Cannon-Albright, Hermann Brenner, Jong Park, Radka Kaneva, Jyotsna Batra, Manuel R. Teixeira, Hardev Pandha, Mark Lathrop, George Davey Smith, The PRACTICAL consortium

**Affiliations:** School of Social and Community Medicine, University of Bristol, Bristol, UK; MRC Integrative Epidemiology Unit at the University of Bristol, Bristol, UK; National Institute for Health Research, Bristol Biomedical Research Unit in Nutrition, Bristol, UK; Nuffield Department of Surgery, University of Oxford, Oxford, UK; Surgical Oncology (Uro-Oncology: S4), University of Cambridge, Box 279, Addenbrooke’s Hospital, Hills Road, Cambridge, UK; The Institute of Cancer Research, 15 Cotswold Road, Sutton, SM2 5NG Surrey UK; The Royal Marsden NHS Foundation Trust, Fulham and Sutton London and Surrey, UK; Centre for Cancer Genetic Epidemiology, Department of Public Health and Primary Care, University of Cambridge, Strangeways Research Laboratory, Worts Causeway Cambridge, UK; University of Warwick, Coventry, UK; Institute of Population Health, The University of Manchester, Manchester, M13 9PL UK; The Cancer Council Victoria, 615 St. Kilda Road, Melbourne, Victoria 3004 Australia; Centre for Epidemiology and Biostatistics, Melbourne School of Population and Global Health, The University of Melbourne, Melbourne, Victoria 3010 Australia; Department of Medical Epidemiology and Biostatistics, Karolinska Institute, Stockholm, Sweden; Department of Preventive Medicine, Keck School of Medicine, University of Southern California/Norris Comprehensive Cancer Center, Los Angeles, CA USA; Department of Medical Biochemistry and Genetics, University of Turku, Turku, Finland; Institute of Biomedical Technology/BioMediTech, University of Tampere and FimLab Laboratories, Tampere, Finland; Department of Clinical Biochemistry, Herlev Hospital, Copenhagen University Hospital, Herlev Ringvej 75, Herlev, DK-2730 Denmark; Cancer Epidemiology Unit, Nuffield Department of Clinical Medicine, University of Oxford, Oxford, UK; Centre for Cancer Genetic Epidemiology, Department of Oncology, University of Cambridge, Strangeways Research Laboratory, Worts Causeway Cambridge, UK; Department of Applied Health Research, University College London, 1-19 Torrington Place, London, WC1E 7HB UK; Cambridge Institute of Public Health, University of Cambridge, Forvie Site, Robinson Way, Cambridge, CB2 0SR UK; Division of Public Health Sciences, Fred Hutchinson Cancer Research Center, Seattle, WA USA; Department of Epidemiology, School of Public Health, University of Washington, Seattle, WA USA; International Epidemiology Institute, 1455 Research Blvd., Suite 550, Rockville, MD 20850 USA; Mayo Clinic, Rochester, MN USA; Department of Urology, University Hospital Ulm, Ulm, Germany; Institute of Human Genetics, University Hospital Ulm, Ulm, Germany; Brigham and Women’s Hospital/Dana-Farber Cancer Institute, 45 Francis Street - ASB II-3, Boston, MA 02115 USA; Washington University, St Louis, MO USA; International Hereditary Cancer Center, Department of Genetics and Pathology, Pomeranian Medical University, Szczecin, Poland; Division of Genetic Epidemiology, Department of Medicine, University of Utah School of Medicine, Salt Lake City, UT USA; Division of Clinical Epidemiology and Aging Research, German Cancer Research Center (DKFZ), Heidelberg, Germany; Division of Preventive Oncology, German Cancer Research Center (DKFZ), Heidelberg, Germany; German Cancer Consortium (DKTK), German Cancer Research Center (DKFZ), Heidelberg, Germany; Division of Cancer Prevention and Control, H. Lee Moffitt Cancer Center, 12902 Magnolia Dr., Tampa, FL USA; Molecular Medicine Center and Department of Medical Chemistry and Biochemistry, Medical University-Sofia, 2 Zdrave St., Sofia, 1431 Bulgaria; Australian Prostate Cancer Research Centre – Qld, Institute of Health and Biomedical Innovation and School of Biomedical Sciences, Queensland University of Technology, Brisbane, Australia; Department of Genetics, Portuguese Oncology Institute, Porto, Portugal; Biomedical Sciences Institute (ICBAS), Porto University, Porto, Portugal; The University of Surrey, Guildford, Surrey GU2 7XH UK; Commissariat à l’Energie Atomique, Center National de Génotypage, Evry, France; McGill University-Génome Québec Innovation Centre, Montreal, Canada

**Keywords:** Boys, Mendelian randomization, Prostate cancer, Puberty, Tanner scale

## Abstract

**Background:**

Epidemiological studies have observed a positive association between an earlier age at sexual development and prostate cancer, but markers of sexual maturation in boys are imprecise and observational estimates are likely to suffer from a degree of uncontrolled confounding. To obtain causal estimates, we examined the role of pubertal development in prostate cancer using genetic polymorphisms associated with Tanner stage in adolescent boys in a Mendelian randomization (MR) approach.

**Methods:**

We derived a weighted genetic risk score for pubertal development, combining 13 SNPs associated with male Tanner stage. A higher score indicated a later puberty onset. We examined the association of this score with prostate cancer risk, stage and grade in the UK-based ProtecT case-control study (*n* = 2,927), and used the PRACTICAL consortium (*n* = 43,737) as a replication sample.

**Results:**

In ProtecT, the puberty genetic score was inversely associated with prostate cancer grade (odds ratio (OR) of high- vs. low-grade cancer, per tertile of the score: 0.76; 95 % CI, 0.64–0.89). In an instrumental variable estimation of the causal OR, later physical development in adolescence (equivalent to a difference of one Tanner stage between pubertal boys of the same age) was associated with a 77 % (95 % CI, 43–91 %) reduced odds of high Gleason prostate cancer. In PRACTICAL, the puberty genetic score was associated with prostate cancer stage (OR of advanced vs. localized cancer, per tertile: 0.95; 95 % CI, 0.91–1.00) and prostate cancer-specific mortality (hazard ratio amongst cases, per tertile: 0.94; 95 % CI, 0.90–0.98), but not with disease grade.

**Conclusions:**

Older age at sexual maturation is causally linked to a reduced risk of later prostate cancer, especially aggressive disease.

**Electronic supplementary material:**

The online version of this article (doi:10.1186/s12916-016-0602-x) contains supplementary material, which is available to authorized users.

## Background

Prostate cancer is now the most frequently detected cancer among men in Westernized countries [1]. Prostatic intraepithelial neoplasia, a precursor of prostate cancer, has been observed among men in their 20s, suggesting that early-life exposures may play a role in the development of prostate cancer [2] and provide novel opportunities for prostate cancer prevention [3].

Circulating hormones, which rise during puberty, in particular androgens and insulin-like growth factors (IGFs), may play a role in prostate cancer initiation and progression [4, 5], although the relevance of serum androgen levels has recently been called into question [6]. Age at onset of puberty may be a risk factor for prostate adenocarcinoma in men given that exposure to high levels of hormones takes place during the critical window of prostate development in adolescence [3]. Age of menarche is a well-known risk factor for breast cancer [7], but it is yet unclear whether sexual maturation similarly influences later life cancer events in men. However, timing of puberty in boys is difficult to measure as it is not defined by a specific event as in women (menarche); thus, assessing it as a risk factor for prostate cancer in men is challenging.

We investigated whether pubertal development influences risk of prostate cancer in a population-based cohort. We used a genetic score comprised of single nucleotide polymorphisms (SNPs) associated with Tanner genital stage in adolescent boys [8, 9], as a surrogate for the onset and progression of pubertal changes, and we determined associations of this genetic score with prostate cancer risk, stage and grade. The Tanner scale is a widely used 5-point scale that rates breast development in girls, genital development in boys, and pubic hair growth in both [10]. Using a genetic score instead of directly assessed Tanner stage, in an approach known as Mendelian randomization (MR) [11], allows stronger causal inferences because genetic variants are usually unaffected by non-genetic confounding, reverse causality, or measurement error, which underlie the problematic interpretation of observational studies [11, 12].

## Methods

### Subjects

This is a case-control study nested within a multicenter randomized controlled trial of treatments for prostate-specific antigen (PSA)-detected prostate cancer: the Prostate Testing for cancer and Treatment (ProtecT) study (ISRCTN20141297) [13]. During recruitment to the ProtecT study between 2001 and 2009, over 100,000 men aged 50–69 years at 337 general practices in nine UK centres (Birmingham, Bristol, Cambridge, Cardiff, Edinburgh, Leeds, Leicester, Newcastle, Sheffield) were offered a PSA test at a community-based ‘prostate check clinic’, and those with raised levels (≥3 ng/mL) were offered a diagnostic biopsy [14]. Detected tumours were all histologically confirmed and clinically staged using the TNM system [15]. In the current analysis, cancer stages T1-T2 were categorized as ‘localized’; and T3-T4 as ‘locally advanced’, because few tumors had metastasized. Histologic material obtained at biopsy was assigned a Gleason score by specialist uropathologists following a standard proforma and, for the purposes of this study, categorized as low- (score ≤6) or high-grade (score ≥7) cancers. All men without evidence of prostate cancer were eligible for selection as controls; that is, men with a PSA <3 ng/mL or a raised PSA (≥3 ng/mL) combined with at least one negative biopsy and no subsequent prostate cancer diagnosis during the follow-up protocol. We selected one stratum-matched control for each case from those men who had provided a non-fasting blood sample at the prostate check clinic. Controls were randomly selected from the same stratum, i.e. 5-year age-band (age at PSA test) and GP/family practice, as cases.

The working dataset consisted of 2,927 individuals (1,136 cases, 1,791 controls) of European descent with available genotype and phenotype information. All men provided written informed consent prior to inclusion in the study. Trent Multicentre Research Ethics Committee (MREC) approved the ProtecT study (MREC/01/4/025) and the associated ProMPT study which collected biological material (MREC/01/4/061; see Additional file 1: Supplementary Methods for further details).

### Genetic risk score

We derived a genetic risk score for pubertal development in boys based on associations between 13 SNPs and Tanner genital stage in males between 12.6 and 15 years of age described in two recent genome-wide association studies (GWAS) of sexual maturation [8, 9]. All SNPs in the score were associated with Tanner stage in boys (independently of whether they were also associated with Tanner stage in girls or in a combined sample of boys and girls), and they had previously been associated with age at menarche [8, 16], although not always in the direction consistent with their association with Tanner genital stage [9]. Variants in the same gene were included in the score provided their linkage disequilibrium, r^2^, was lower than 0.8.

Scores are used instead of individual genetic variants because they are likely to explain a larger proportion of trait variability and therefore represent stronger proxies for the exposure [12]. Scores were calculated by summing up the dosages of the risk alleles at all 13 SNPs in each individual, weighted by the effect size of the variant in males as reported in the discovery GWAS [8, 9], in such a way that a unit increase in the score corresponded approximately to one risk allele. Risk alleles were those associated with a lower Tanner stage (i.e. delayed pubertal development). Polymorphisms included in the score are shown in Table 1.

**Table 1:** SNPs included in the pubertal development genetic risk score in the ProtecT study

SNP	Nearest gene (distance)	Chr	Position^a^	Tanner stage decreasing/other allele	Tanner stage decreasing allele frequency (ProtecT controls)^b^	Tanner stage decreasing allele frequency (CEU)	Hardy–Weinberg equilibrium *P* value	Gene function
rs2274465	*KDM4A*	1	44121557	C/G	0.664	0.676	0.9	Histone demethylation
rs6427782	*NR5A2* (+198 kb)	1	199798339	A/G	0.510	0.556	0.005	DNA binding/steroid hormone receptor activity
rs6762477	*RBM6*	3	50093209	A/G	0.547	0.551	0.6	Regulation of alternative splicing
rs2153127	*LIN28B* (+36 kb)	6	105348544	T/C	0.530	0.515	0.8	Cell reprogramming
rs7759938	*LIN28B* (+6 kb)	6	105378954	C/T	0.318	0.373	1.0	Cell reprogramming
rs7821178	*PEX2* (–181 kb)	8	78093837	C/A	0.665	0.658	0.1	Peroxisome biogenesis
rs10453225	*TMEM38B* (–381 kb)	9	108920220	G/T	0.681	0.700	0.2	Maintenance of intracellular calcium release
rs2090409	*TMEM38B* (–428 kb)	9	108967088	C/A	0.680	0.688	0.3	Maintenance of intracellular calcium release
rs10739221	*TMEM38B* (–522 kb)	9	109060830	C/T	0.772	0.770	0.5	Maintenance of intracellular calcium release
rs1324913	*KLF12*	13	74635588	T/G	0.338	0.312	0.6	Transcription factor/gene expression regulation
rs12915845	*DET1*	15	89042467	C/T	0.587	0.582	0.1	Development regulation
rs246185	*MKL2* (–35 kb)	16	14395432	C/T	0.313	0.300	0.6	Regulation of immediate early genes/muscle genes
rs12446632	*GPRC5B* (–38 kb)	16	19935389	A/G	0.146	0.127	0.8	Modulation of insulin secretion

^a^Position based on GRCh37.p13 assembly

^b^ *n* = 1,791

### Statistical analysis

Associations of individual SNPs and the multiple SNP score for pubertal development with case/control status and other binary outcomes (localized [reference] vs. locally advanced stage and low [reference] vs. high grade) were determined using logistic regression, with adjustment for age, study center, and the 10 principal components which defined the population structure.

The genetic score was entered into the regression models as a categorical variable with three levels (tertiles). We also used this variable to test for linearity of effect and compare prostate cancer risk among men in the lowest and highest tertiles (i.e. with the earliest and latest sexual maturation, respectively). All analyses were carried out in Stata 13 (StataCorp LP, College Station, TX).

We plotted the effect of each SNP in the genetic score on Tanner stage in approximately 13- to 15-year-old boys against the corresponding effect on high-grade prostate cancer, the disease outcome with the strongest association with the score. The likelihood of bias due to overall directional pleiotropy was formally evaluated with MR-Egger regression [17]. MR-Egger regression also provides an unbiased effect estimate (see definitions in Additional file 1: Supplementary Methods).

We did not have data on Tanner stage measured in adolescence in ProtecT men, and therefore could not estimate its association with the genetic score in ProtecT, in order to run a typical instrumental variable analysis. However, we used a recently developed MR method (summarized data allele score with correlated variants) that provides an estimate of the causal effect of the exposure (i.e. Tanner stage) on the outcome of interest (i.e. prostate cancer) using information on the association of individual SNPs in the score with exposure and outcome [18]. We obtained the effect estimates of SNPs on Tanner stage at approximately 13–15 years from published GWAS data [8, 9].

Replication analyses were carried out in the PRACTICAL consortium (PRostate cancer AssoCiation group To Investigate Cancer-Associated aLterations in the genome) to test the association of the puberty genetic score with prostate cancer risk and progression. Overall, there were 45,928 individuals of European ancestry, of which 22,160 prostate cancer cases and 21,577 controls had genotype data available after applying quality control procedures. Additionally, amongst men with prostate cancer in PRACTICAL, we estimated associations of the puberty score with 15-year all-cause and prostate cancer-specific mortality (as an indication of long-term survival) using Cox proportional hazards regression with date at diagnosis as the start date and date at death or final follow-up as the exit date, adjusted for age at diagnosis and 15 principal components, with robust standard errors to account for within study clustering. All studies in the consortium have the relevant Institutional Review Board approval in each country in accordance with the Declaration of Helsinki. More detailed information is provided in the consortium website (http://practical.ccge.medschl.cam.ac.uk) and Additional file 1: Supplementary Methods and Tables S1 and S2.

## Results

### ProtecT

Men with prostate cancer were on average older, had less benign prostatic hyperplasia (BPH), a lower body mass index (BMI), more relatives with prostate cancer, lower IGF-I, and higher IGF-II and IGF binding protein (BP)-3 levels than controls (Table 2). The IGF-I:IGFBP-3 molar ratio, an indicator of bioavailable IGF-I, was consequently lower in patients; 30 % of men with prostate cancer were classified as having high-grade disease (Gleason score ≥7), and 12 % as having locally advanced disease (TNM stages T3-T4).

**Table 2:** Clinical characteristics of prostate cancer cases and controls in the ProtecT study

	Cases	Controls	n	*P* value
Total, n	1,136	1,791	2,927	
Age, years	62.2 ± 5.1	61.6 ± 5.2	2,927	0.003
PSA, ng/mL	8.5 ± 15.4	1.1 ± 1.3	2,925	<0.001
Gleason grade, % (<7/≥7)	70.0/30.0	n/a	1,135	
TNM stage, % (localized/advanced)	88.4/11.6	n/a	1,136	
BPH, % (no/yes)^a^	92.7/7.3	89.0/11.0	1,363	0.02
BMI, kg/m^2^	27.0 ± 3.7	27.5 ± 4.1	1,973	0.01
Height, cm	176.4 ± 7.0	175.9 ± 6.9	2,078	0.2
Weight, kg	84.5 ± 13.0	85.9 ± 14.6	2,677	0.02
leg length, cm	76.7 ± 4.8	76.5 ± 4.6	2,055	0.3
Birthweight, g	3,437.2 ± 744.9	3,476.1 ± 663.4	939	0.4
Family history, % (no/yes)^b^	92.7/7.3	95.0/5.0	2,908	0.01
Diabetes, % (no/yes)^c^	92.8/7.2	91.1/8.9	1,895	0.2
IGF-I, ng/mL	156.1 ± 55.8	163.2 ± 57.0	1,756	0.01
IGF-II, ng/mL	862.1 ± 323.6	733.7 ± 265.3	1,720	<0.001
IGFBP-2, ng/mL	731.5 ± 426.0	726.2 ± 444.7	1,745	0.5^d^
IGFBP-3, ng/mL	4,673.6 ± 1,041.9	4,370.8 ± 1,055.7	1,711	<0.001
IGF-I:IGFBP-3 molar ratio^e^	0.12 ± 0.04	0.14 ± 0.06	1,711	<0.001^d^

Continuous variables: mean ± SD

Two-sided *t*-tests and χ^2^ tests were used to analyze continuous and categorical variables, respectively

*PSA* Prostate-specific antigen, *BMI* Body mass index, *BPH* Benign prostatic hyperplasia, *IGF* Insulin-like growth factor, *IGFBP* Insulin-like growth factor binding protein

^a^n cases = 682, n controls = 681

^b^n cases = 1,131, n controls = 1,777

^c^n cases = 735, n controls = 1,160

^d^ *P* value obtained from analysis of natural log transformed variable

^e^IGF-I:IGFBP-3 molar ratio = 0.13*[IGF-I]:0.036*[IGFBP-3]

The genetic score was normally distributed (Additional file 1: Figure S1) and for the most part not correlated with population stratification axes (data not shown).

We found evidence of an inverse association between our genetic score for pubertal development in boys and prostate cancer, i.e. the higher the score and, thus, the later the sexual maturation, the lower the risk for prostate cancer. The association was particularly strong for Gleason grade (odds ratio (OR) low- vs. high-grade disease, per tertile: 0.76; 95 % CI, 0.64–0.89; *P* = 0.001; Table 3). A dose-response effect of the genetic score in tertiles on high-grade prostate cancer was observed. Men in the highest score tertile (representing the most sexually immature individuals at a specific age) had a 43 % (95 % CI, 21–59 %) lower risk of high- versus low-grade disease than men in the lowest tertile (Table 4).

**Table 3:** Pubertal development genetic risk score and prostate cancer risk, stage and grade in the ProtecT study

Trait	n	OR	95 % CI	*P* value
Control/case (0/1)	2,927	0.95	0.87–1.04	0.3
Gleason score (0:≤6/1:≥7)^a^	1,135	0.76	0.64–0.89	0.001
Stage (0:localised/1:locally advanced)	1,136	0.80	0.64–1.01	0.06
BPH (0:no/1:yes)	1,363	1.11	0.88–1.40	0.4

ORs indicate effects per tertile increase in the genetic score, adjusted by age, recruitment centre and 10 principal components for population structure

^a^0 corresponds to the reference category

*BPH* Benign prostatic hyperplasia, *CI* Confidence intervals, *OR* Odds ratio

**Table 4:** Odds ratios (ORs) for high- vs low-grade prostate cancer by pubertal development genetic risk score tertiles in the ProtecT study

Genetic score tertiles	OR	95 % CI	*P* value
T1	Reference		0.003
T2	0.79	0.58–1.07	
T3	0.57	0.41–0.79	

High-grade prostate cancer = Gleason ≥7

Low-grade prostate cancer = Gleason ≤6

OR adjusted by age, recruitment centre and 10 principal components for population structure

*n* = 1,135

*CI* Confidence intervals

**Fig. 1:**
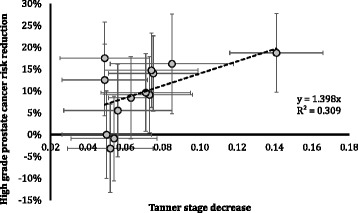
Effect of Tanner stage change in boys on the risk of developing high-grade prostate cancer. In ProtecT, proportional risk reduction for high-grade prostate cancer (Gleason ≥7) for each SNP plotted against each SNP’s absolute effect on lowering Tanner stage. The trend line, set to intercept the axes at the origin, represents the percentage risk reduction for high-grade disease per unit decrease in Tanner stage. Excluding SNP rs6427782, which was out of Hardy–Weinberg equilibrium pre-Bonferroni correction for multiple testing, from the plot did not produce an appreciable change in the results. Tanner genital stage in boys was treated as a quantitative trait on a scale of 1–5, according to the studies where the associated SNPs were first described [8, 9]

The reported effect of each SNP in the score on Tanner stage in boys [8, 9] was correlated with the corresponding effect on having high-, compared to low-grade, prostate cancer (R^2^ ~ 31 %; Additional file 1: Table S3, Fig. 1).

Overall, there was no evidence of an association between the genetic score and potential confounders among controls, such as age, BMI, weight, birthweight, BPH, family history of prostate cancer, or diabetes. No association between the genetic score and PSA was found either. Marginal positive associations with leg length, a trait affected by the timing of puberty [19], and adult height were detected. In addition, we uncovered weak associations with IGFBP-2 and IGFBP-3 serum levels, as well as with the IGF-I:IGBP-3 molar ratio (Additional file 1: Table S4).

Using an estimated genetic score with summarized data [18] we determined that there would be a substantial reduction in high-grade (compared to low-grade) prostate cancer per unit decrease in Tanner stage in relation to peers (OR: 0.23; 95 % CI, 0.09–0.57; *P* = 0.002). The MR-Egger’s test did not suggest the presence of directional pleiotropy (*P* for intercept >0.05; see symmetrical funnel plot in Additional file 1: Figure S2), and gave a similar causal estimate to that obtained with the allele score with the summarized data method (OR: 0.16; 95 % CI, 0.04–2.94; *P* = 0.2). No heterogeneity was apparent in the causal estimates obtained from each genetic variant individually (I^2^ = 0.0 %, *P* = 1.0).

### PRACTICAL

We created a weighted genetic score with 12 of the 13 SNPs used in ProtecT, as rs1324913 was not available in PRACTICAL. Information on SNPs in the score is provided in Additional file 1: Table S5, and the score distribution is shown in Additional file 1: Figure S1. No correlation between the Tanner score and principal components was evident, and similarly, the score was not associated with age at diagnosis, family history, or method of disease detection (not shown).

**Fig. 2:**
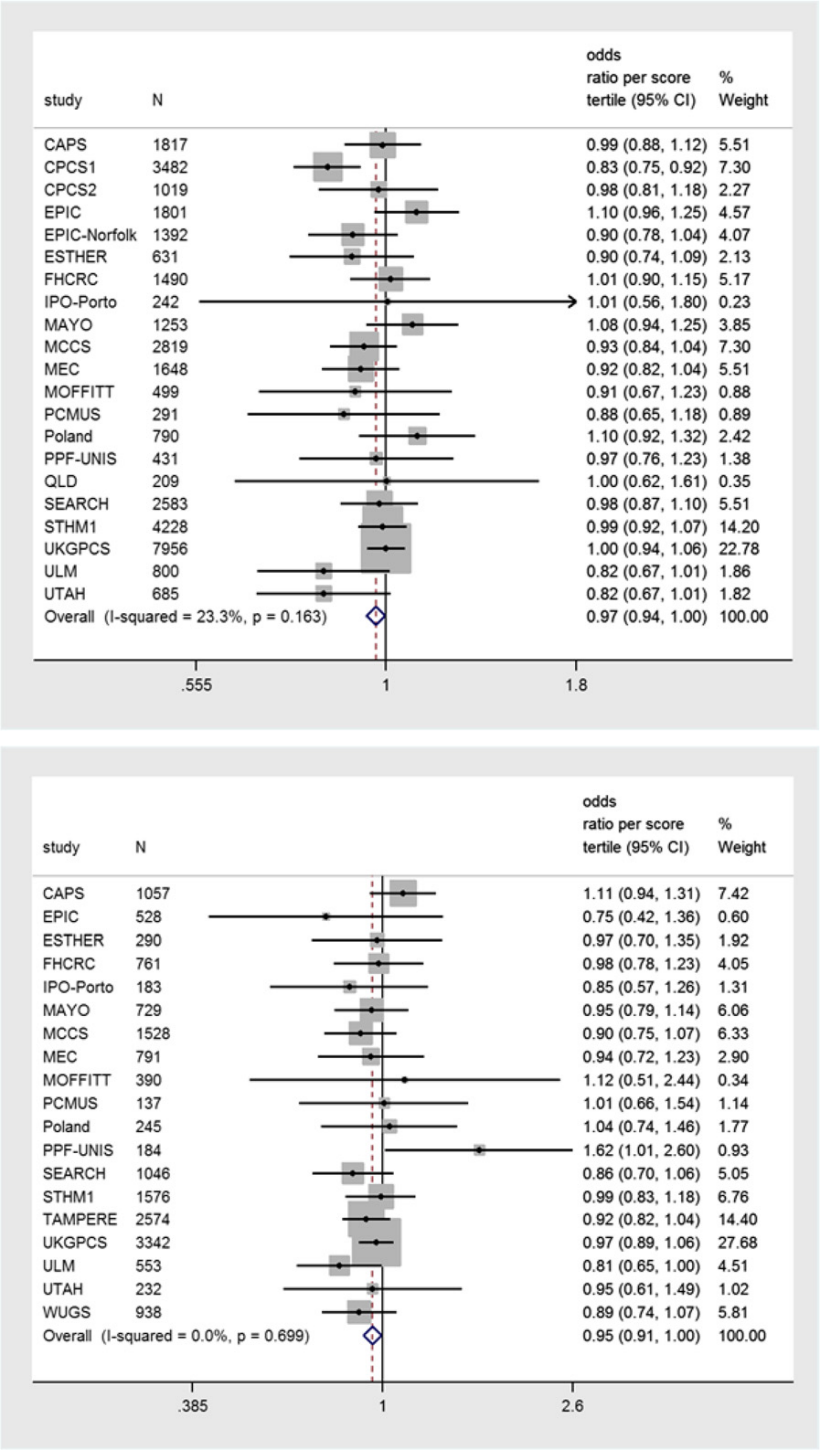
Pubertal development genetic risk score and prostate cancer risk (top) and stage (bottom) in the PRACTICAL consortium

In a meta-analysis of 21 studies included in PRACTICAL, the genetic score was associated with prostate cancer risk, such that a higher score – and therefore, a delayed maturation – showed a protective effect (OR per tertile: 0.97; 95 % CI, 0.94–1.00; *P* = 0.03). A slightly bigger effect was found with prostate cancer stage (localized vs. advanced, OR per tertile: 0.95; 95 % CI, 0.91–1.00; *P* = 0.03; Fig. 2) but not with grade (low vs. high grade, OR per tertile: 0.98; 95 % CI, 0.95–1.02; *P* = 0.4; Additional file 1: Table S6). Heterogeneity between studies was low (I^2^ < 33 %; *P* >0.05).

There were 15 studies with mortality data in PRACTICAL. Overall, the average time to death or final follow-up was 7 years, with a maximum ranging from approximately 6 (in PCMUS) to 38 years (in Tampere). The genetic score was associated with 15-year prostate cancer-specific mortality amongst men with prostate cancer (hazard ratio (HR) per tertile: 0.94; 95 % CI, 0.90–0.98; *P* = 0.01), and marginally with 15-year all-cause mortality (HR per tertile: 0.97; 95 % CI, 0.95–1.00; *P* = 0.04). The proportional hazards assumption was not met (*P* <0.001), probably due to the fact that up to 5 years post-diagnosis there do not appear to be differences in survival between individuals with different numbers of risk alleles (i.e. alleles associated with later pubertal timing; Additional file 1: Figure S3).

We estimated the effect of being ranked a unit lower in the Tanner stage (for the same age) on 15-year prostate cancer-specific mortality as HR 0.62 (95 % CI, 0.49–0.78; *P* <0.001). The corresponding funnel plot and MR-Egger results, which did not uncover evidence of pleiotropy, are shown in Additional file 1: Figure S4.

## Discussion

### Main findings

In a study of PSA-detected prostate cancer cases and controls we found strong evidence that a genetic score, comprised of SNPs associated with Tanner stage in approximately 13- to 15-year-old boys, was inversely associated with prostate cancer progression. A later pubertal development (expected among those with higher genetic score values) lowered the risk of developing high-grade disease, a possible clinically relevant subtype because of its stronger relationship than low-grade disease to progression.

Replication analysis using 21 prostate cancer studies across Europe, the USA and Australia in the PRACTICAL consortium, uncovered a weak inverse association between prostate cancer risk and stage and the puberty genetic score, with a reduced effect detected on disease grade. However, we found a stronger association of the score with prostate cancer-specific mortality up to 15 years after diagnosis, indicating that (on average) men whose sexual maturation was later than their peers were less likely to die due to the disease than those whose onset of puberty was earlier. This is in agreement with our findings in ProtecT, regarding the association of earlier puberty with high-grade disease, as men with more aggressive cancer tend to have a poorer prognosis [20]. It is possible that differing definitions of low- and high-grade prostate cancer across studies may have prevented the detection of an effect of the puberty score on this phenotype, with mortality being a stronger and more clear-cut marker of an aggressive disease. There were also differences between studies in method of disease detection: the cases enrolled in ProtecT were PSA-detected, whereas the majority of men in the PRACTICAL studies were clinically identified. Additionally, in PRACTICAL, there was a wide variation in the proportion of men with a positive family history of prostate cancer, which ranged from as low as 2.4 % in EPIC to 42.4 % in WUGS (conversely, the proportion of men with a positive family history in ProtecT was ~6 %).

### Mechanisms explaining the observed associations

It has been suggested that endogenous androgen and IGF-I hormones may underlie the relationship between puberty timing and prostate cancer risk [4, 5]. The concentrations of these hormones increases markedly during puberty and are likely to be especially influential on the prostate gland as it becomes fully developed at this time [21]. Because the prostate is still maturing, puberty may be an important biological window at which early life exposures could have long-term effects on the prostate [3].

Androgens play a central role in the etiology of prostate cancer, as prostate cancer is dependent on androgen receptor activation for growth and survival [22]. A delayed pubertal onset may reduce the length of time an individual is exposed to high androgen levels during a period when the prostate is particularly susceptible to carcinogenic exposures [3].

IGF-I is a potent mitogen and inhibitor of apoptosis that mediates growth during childhood and adolescence and, consequently, stimulates carcinogenesis. IGF-I levels increase from birth to a pubertal peak before declining steadily from young adulthood [23, 24]. It is possible that a younger age at the initiation of puberty is linked to an increase in prostate cancer risk by its association with higher IGF-I levels. Serum IGF-I has been positively associated with an earlier pubertal age in an observational study examining adult IGF-I levels, suggesting that higher pre-pubertal IGF-I (if reflected by higher adult IGF-I) may accelerate childhood growth and the start of puberty [24].

In our study, the puberty genetic score was not associated with circulating IGF-I, although the effect appeared to be in the direction anticipated [24], but we observed an inverse association with the IGF-I:IGFBP-3 molar ratio, suggesting that an earlier pubertal development may be influenced by higher levels of bioavailable IGF-I.

The weakly positive association of the genetic score with adult leg length and height agrees with studies that showed that earlier age at puberty was associated with shorter stature, primarily attributable to shorter leg length, in US women [25] and Swedish men [19]. Growth in leg length, an indicator of pre-pubertal living conditions, on the other hand, has been positively associated with IGF-I levels in UK children, particularly boys [26].

Our finding that a younger age at sexual maturation increases the risk of developing high-grade prostate cancer, and of dying due to the disease, strengthens the idea of the existence of trade-offs between reproductive success and health. MR analysis suggests that there may be a causal relationship between early life environments that promote an accelerated onset of puberty under conditions of uncertainty (e.g. in cases of familial stress due to low income, marital conflict or father absence), so as to favour reproduction, and a detrimental effect on health and longevity in the long term [27, 28].

### Strengths and limitations

Studies of puberty in men are problematic because its initiation is not defined as a single event in the way that menarche is. In addition, in studies of middle aged and elderly men, an attempt to measure puberty is likely to suffer from recall bias. The genetic score represents a more accurate instrument to assess the causality of the association of pubertal development and prostate cancer risk. The association with leg length provides to some extent a validation of the genetic score in the ProtecT population.

A genetic score is unlikely to be associated with non-genetic confounders, which frequently obscure the interpretation of observational data, and this is, in fact, the case in our study with respect to a few measured confounders.

As the genetic score in our study was not associated with age, PSA, BMI, diabetes, or BPH, we believe that its association with prostate cancer does not represent an artefact of detection due to, for instance, men who are seen more frequently by a doctor having an incidental diagnosis of prostate cancer.

One important assumption in MR is that the instrument (i.e. the genetic score) should be associated with the outcome of interest (i.e. prostate cancer) only via the exposure (i.e. pubertal development). Some SNPs in the score have been associated with height *(P* <0.05, http://www.gwascentral.org/index) while four of them are located near genes (*LIN28B* and *TMEM38B*) recently associated with sitting height ratio and found to disproportionately affect leg length [29]. However, this could be an example of mediated pleiotropy (where a single process influences a cascade of events) [30] and as such it does not undermine our findings. Furthermore, a formal test of the assumption of no pleiotropy, implemented using MR-Egger’s regression, found no evidence of a violation of this principle.

Replication of our findings as well as uncovering the potential mechanisms through which the timing of puberty might affect the progression of prostate cancer were likely hindered by differences in phenotype ascertainment in PRACTICAL studies.

### Comparison with existing literature

Few studies have examined the role that pubertal development has on the initiation and progression of prostate cancer, in contrast to the more extensive research on age at menarche and breast cancer. This research shows that an earlier age at menarche is reliably associated with greater breast cancer risk [31]. Given the difficulties in defining puberty among males, studies have used a variety of traits as proxies, mainly age at different life events such as shaving initiation [21, 32], first sexual intercourse [33–36], first ejaculation [37], peak height velocity [24], attainment of adult height [38], as well as the well-established Tanner scale [10]. Results from studies that assessed these variables with respect to prostate cancer showed that later growth relative to peers [37], height attainment [38], age at first sexual intercourse [33–36], and being older than 16 at first ejaculation [37] were all associated in a protective direction with prostate cancer. Older age at initiation of shaving was also protective but only among African Americans [21]. As far as we know, no observational study to date has used Tanner stage to investigate the relationship between pubertal development and prostate cancer, so a comparison with our MR findings is not possible.

## Conclusions

Using an MR approach, we have found evidence that experiencing a later sexual maturation reduces prostate cancer risk, especially that of aggressive prostate cancer, as well as mortality owing to the disease. The mechanisms that underlie this relationship may involve the androgenic or IGF pathways, but additional MR studies, using specific instruments for these exposures, should be carried out to investigate this further. Although altering pubertal timing is not a viable prostate cancer prevention strategy there is public health value in identifying those individuals who are more likely to have a worse prognosis [3]. On the other hand, if pubertal development is shown to be driven by increased IGF-I levels, then dietary interventions to regulate its course could potentially be considered.

## Availability of data and materials

Summary data is provided in Additional file 2.

## Additional files

Additional file 1: Tables S1 – S6. Figures S1–S4. Supplementary Methods. Definitions. Supplementary References. (DOCX 214 kb) Additional file 2: Summary data of the association of each SNP in the pubertal development genetic risk score with exposure and outcomes in the PRACTICAL consortium. (XLSX 13 kb)

## Abbreviations

BMI: Body mass index
BPH: Benign prostatic hyperplasia
GWAS: Genome-wide association study
IGF: Insulin-like growth factor
IGFBP: Insulin-like growth factor binding protein
LD: Linkage disequilibrium
MR: Mendelian randomization
PSA: Prostate-specific antigen
SNP: Single nucleotide polymorphism
