# Supplementary material for: Pubertal development and prostate cancer risk: Mendelian randomization study in a population-based cohort
Source: BMC Med. 2016 Apr 4;14:66. doi: 10.1186/s12916-016-0602-x (PMC4820939; doi:10.1186/s12916-016-0602-x)
Supplement: Additional file 1: Tables S1 – S6. — Figures S1–S4. Supplementary Methods. Definitions. Supplementary References. (DOCX 214 kb) [file 12916_2016_602_MOESM1_ESM.docx]

**Table S1. Clinical characteristics of prostate cancer cases in 25 PRACTICAL studies. N = 45,928 men.**

| **study** | **country** | **N controls** | **N cases** | **mean age at diagnosis (years)** | **mean PSA at diagnosis (ng/ml)** | **European ethnicity (%)^a^** | **family history of prostate cancer (%)^a,b^** | **high Gleason score (≥7, %)^a^** | **advanced stage (%)^a,c^** | **screen-detected cancer (%)^a^** |
| --- | --- | --- | --- | --- | --- | --- | --- | --- | --- | --- |
| **CAPS** | Sweden | 664 | 1153 | 66.1 | 79.6 | 100 | 17.4 | 49.9^f^ | 30.3 | 0.0 |
| **CPCS1** | Denmark | 2756 | 848 | 69.5 | 48.0 | 99.6 | 8.2^f^ | 71.2^f^ | n/a | 0.0 |
| **CPCS2** | Denmark | 1001 | 265 | 64.9 | 36.0 | 99.4 | 14.7^f^ | 52.2^f^ | n/a | 0.0 |
| **EPIC** | Europe | 1079 | 722 | 64.9 | 0.2 | 100 | n/a | 27.9^f^ | 4.0^f^ | 0.0 |
| **EPIC-Norfolk** | UK | 911 | 481 | 72.1 | n/a | 99.9 | 2.5 | 39.4^f^ | n/a | n/a |
| **ESTHER** | Germany | 318 | 313 | 65.5 | 58.7 | 100 | 8.9^f^ | 48.0 | 27.6 | 61.9^f^ |
| **FHCRC** | USA | 729 | 761 | 59.7 | 16.1 | 99.9 | 21.7 | 41.7 | 20.2 | n/a |
| **IPO-Porto** | Portugal | 66 | 183 | 59.3 | 8.3 | 100 | 20.0^f^ | 84.2 | 64.5 | 82.8^f^ |
| **MAYO** | USA | 488 | 767 | 65.2 | 15.5 | 100 | 29.1 | 55.3^f^ | 45.5 | 73.7^f^ |
| **MCCS^d^** | Australia | 1169 | 1650 | 58.5 | 18.8 | 98.8 | 23.5^f^ | 53.4 | 14.5 | n/a |
| **MEC** | USA | 829 | 819 | 69.5 | n/a | 100 | 13.0 | n/a | 12.5 | n/a |
| **MOFFITT** | USA | 96 | 404 | 65.0 | 7.3 | 97.5 | 22.3 | 43.4 | 3.6 | 0.0^f^ |
| **PCMUS** | Bulgaria | 140 | 151 | 69.3 | 32.5 | 100 | 5.3 | 59.6 | 46.7 | 21.2 |
| **Poland** | Poland | 359 | 438 | 67.7 | 40.2 | 100 | 10.6 | 32.8^f^ | 37.1^f^ | 0.0^f^ |
| **PPF-UNIS** | UK | 187 | 244 | 68.9 | 32.1 | 99.8 | 25.3 | 45.2^f^ | 28.8^f^ | n/a |
| **ProMPT** | UK | 2 | 166 | 66.3 | 33.0 | 100 | 34.6 | 74.3^f^ | 34.7 | 0.0^f^ |
| **ProtecT** | UK | 1458 | 1545 | 62.7 | 9.6 | 99.7 | 8.0^f^ | 29.9 | 11.4 | 100.0 |
| **QLD** | Australia | 85 | 139 | 61.4 | 7.4 | 99.1^e^ | 37.8 | 83.6 | 0.0^f^ | n/a |
| **SEARCH** | UK | 1231 | 1354 | 63.1 | 53.2 | 100 | 16.3 | 56.9^f^ | 18.0^f^ | 36.7^f^ |
| **STHM1** | Sweden | 2224 | 2006 | 66.2 | n/a | 100 | 20.2 | 45.5^f^ | 14.4^f^ | n/a |
| **TAMPERE** | Finland | 2413 | 2754 | 68.2 | 69.2 | 100 | n/a | 43.8^f^ | 21.4 | 46.8 |
| **UKGPCS** | UK | 4132 | 3838 | 63.6 | 88.0 | 99.8 | 22.4^f^ | 50.5^f^ | 36.4^f^ | 28.0^f^ |
| **ULM** | Germany | 354 | 603 | 63.8 | 19.1 | 100 | 44.9 | 51.3^f^ | 40.5 | n/a |
| **UTAH** | USA | 245 | 440 | 62.6 | n/a | 100 | 51.4 | n/a | 17.2^f^ | n/a |
| **WUGS** | USA | 0 | 948 | 60.8 | 6.1 | 95.8 | 42.6^f^ | 59.3 | 24.2 | n/a |

Information in the table is given for the subset of individuals whose ethnicity was “European” (except for the study’s European ethnicity percentage).

^a^Percent of cases with data available.

^b^Family history of prostate cancer in a first degree relative.

^c^T3 or T4 on TNM staging, or if not available, “regional” or “distant” on SEER staging.

^d^MCCS includes studies Risk Factors for Prostate Cancer Study (RFPCS) and The Early Onset Prostate Cancer Study (EOPCS).

^e^Information missing for more than 10% of individuals.

^f^Information missing for more than 10% of patients.

n/a not available

**Table S2. Ethics committees for the studies participating in the PRACTICAL consortium.**

| **study** | **ethics committee** |
| --- | --- |
| CAPS | Forskningsetikkommittén vid Karolinska Institutet (regional) |
| CPCS1 | Videnskabsetiske Komité D for Region Hovedstaden |
| CPCS2 | Videnskabsetiske Komité D for Region Hovedstaden |
| EPIC | IARC Ethics Committee (IEC) |
| EPIC-Norfolk | Norwich District Ethics Committee |
| ESTHER | Ethikkommission der Medizinische Fakultät Heidelberg |
| FHCRC | Fred Hutchinson Cancer Research Center Institutional Review Board |
| IPO-Porto | Comissão de Ética-IPO Porto |
| MAYO | MAYO Clinic Institutional Review Board |
| MCCS | The Cancer Council Victoria Human Research Ethics Committee |
| MEC | University of Southern California Health Sciences Institutional Review Board |
| MOFFITT | University of South Florida Institutional Review Board |
| PCMUS | Medical University-Sofia Ethics Committee for Scientific Research |
| Poland | Pomorska Akademia Medyczna Komisja Bioetyczna |
| PPF-UNIS | National Research Ethics Service Leeds (East) Research Ethics Committee |
| ProMPT | Trent Multi-centre Research Ethics Committee |
| ProtecT | Trent Multi-centre Research Ethics Committee |
| QLD | The Queensland Institute of Medical Research Human Research Ethics Committee |
| SEARCH | National Research Ethics Service Cambridgeshire 4 Research Ethics Committee |
| STHM1 | Forskningsetikkommittén vid Karolinska Institutet (regional) |
| TAMPERE | Tampere University Hospital Institutional Review Board/City of Tampere Institutional Review Board/The Ministry of Health and Social Affairs/National Authority for Medical Affairs |
| UKGPCS | London Muti-centre Research Ethics Committee/Trent Research Ethics Committee |
| ULM | Ethikkommission der Universität Ulm |
| UTAH | University of Utah Institutional Review Board |
| WUGS | Washington University Human Research Protection Office (HRPO) |

**Table S3. Tanner stage-associated SNPs and prostate cancer risk (N=2927), Gleason grade (N=1135) and disease stage (N=1136), in the ProtecT study.**

| **SNP** | **OR**  **(cases vs controls)** | **95%CI** | **p-value** | **OR Gleason (≥ 7 vs ≤ 6)** | **95%CI** | **p-value** | **OR stage**  **(locally advanced vs localized)** | **95% CI** | **p-value** |
| --- | --- | --- | --- | --- | --- | --- | --- | --- | --- |
| rs10453225 | 0.98 | (0.88,1.10) | 0.73 | 0.85 | (0.70,1.04) | 0.1 | 0.85 | (0.64,1.12) | 0.2 |
| rs10739221 | 0.96 | (0.84,1.09) | 0.52 | 0.95 | (0.76,1.18) | 0.6 | 0.74 | (0.55,1.01) | 0.1 |
| rs12446632 | 1.03 | (0.89,1.20) | 0.71 | 0.84 | (0.64,1.09) | 0.2 | 0.89 | (0.61,1.30) | 0.5 |
| rs12915845 | 0.88 | (0.79,0.98) | 0.02 | 0.91 | (0.75,1.10) | 0.3 | 1.08 | (0.83,1.41) | 0.6 |
| rs1324913 | 0.97 | (0.87,1.08) | 0.58 | 1.00 | (0.82,1.22) | 1.0 | 0.99 | (0.75,1.32) | 1.0 |
| rs2090409 | 0.98 | (0.88,1.10) | 0.79 | 0.86 | (0.71,1.05) | 0.1 | 0.85 | (0.64,1.12) | 0.2 |
| rs2153127 | 1.01 | (0.90,1.12) | 0.93 | 0.88 | (0.73,1.05) | 0.2 | 1.01 | (0.78,1.32) | 0.9 |
| rs2274465 | 0.96 | (0.86,1.07) | 0.48 | 0.90 | (0.75,1.10) | 0.3 | 0.82 | (0.62,1.07) | 0.1 |
| rs246185 | 0.97 | (0.86,1.10) | 0.67 | 0.81 | (0.65,1.01) | 0.06 | 0.93 | (0.68,1.27) | 0.7 |
| rs6427782 | 0.94 | (0.84,1.04) | 0.24 | 1.03 | (0.85,1.25) | 0.8 | 0.79 | (0.60,1.05) | 0.1 |
| rs6762477 | 0.92 | (0.83,1.03) | 0.14 | 1.01 | (0.84,1.22) | 0.9 | 0.98 | (0.75,1.29) | 0.9 |
| rs7759938 | 1.01 | (0.90,1.13) | 0.87 | 0.92 | (0.75,1.12) | 0.4 | 0.96 | (0.72,1.27) | 0.8 |
| rs7821178 | 0.94 | (0.84,1.06) | 0.31 | 0.83 | (0.68,1.00) | 0.06 | 0.92 | (0.69,1.21) | 0.5 |

ORs indicate per allele effects adjusted by age, recruitment centre and 10 principal components for population structure.

**Table S4. Pubertal development genetic risk score (13 SNPs) and risk factors for prostate cancer in ProtecT controls.**

| **trait** | **N** | **effect** | **95% CI** | **p-value** |
| --- | --- | --- | --- | --- |
| age (years) | 1791 | -0.10 | (-0.39,0.20) | 0.5 |
| PSA (ng/ml)^a^ | 1789 | 0.02 | (-0.03,0.07) | 0.4 |
| BMI (kg/m^2^) | 1181 | 0.04 | (-0.25,0.33) | 0.8 |
| height (cm) | 1256 | 0.47 | (-0.002,0.94) | 0.05 |
| weight (kg) | 1638 | 0.61 | (-0.26,1.47) | 0.2 |
| birthweight (g) | 580 | -34.34 | (-100.42,31.74) | 0.3 |
| family history (0:no/1:yes)^b^ | 1777 | 1.02 | (0.78,1.33) | 0.9 |
| diabetes (0:no/1:yes) | 1160 | 1.03 | (0.80,1.33) | 0.8 |
| leg length (cm) | 1238 | 0.30 | (-0.01,0.61) | 0.06 |
| IGF-I (ng/ml) | 727 | -1.48 | (-6.55,3.60) | 0.6 |
| IGF-II (ng/ml) | 718 | 14.81 | (-8.71,38.33) | 0.2 |
| IGFBP-2 (ng/ml)^a^ | 724 | -0.05 | (-0.11,-0.004) | 0.04 |
| IGFBP-3 (ng/ml) | 712 | 96.15 | (2.24,190.06) | 0.05 |
| IGF-I:IGFBP-3 molar ratio^a,c^ | 712 | -0.03 | (-0.06, -0.004) | 0.03 |
| BPH (0:no/1:yes) | 681 | 0.75 | (0.55,1.03) | 0.08 |

Per tertile effects adjusted by age, recruitment centre and 10 principal components for population structure.

^a^The natural log was used.

^b^Prostate cancer in father or brother.

^c^IGF-I:IGFBP-3 molar ratio = 0.13*[IGF-I]:0.036*[IGFBP-3]

PSA = Prostate Specific Antigen

BMI = Body Mass Index

BPH = Benign Prostatic Hyperplasia

IGF = Insulin-like Growth Factor

IGFBP = Insulin-like Growth Factor Binding Protein

**Table S5. Tanner stage-associated SNPs included in the pubertal development genetic risk score in the PRACTICAL consortium.**

| **SNP** | **chromosome** | **position^a^** | **Tanner stage decreasing/other allele** | **Tanner stage decreasing allele frequency (PRACTICAL controls)^b^** | **Tanner stage decreasing allele frequency (CEU)** | **Hardy-Weinberg equilibrium p-value** |
| --- | --- | --- | --- | --- | --- | --- |
| rs2274465 | 1 | 44121557 | C/G | 0.656 | 0.676 | 0.2 |
| rs6427782 | 1 | 199798339 | A/G | 0.517 | 0.556 | 0.04 |
| rs6762477 | 3 | 50093209 | A/G | 0.565 | 0.551 | 0.5 |
| rs2153127 | 6 | 105348544 | T/C | 0.533 | 0.515 | 0.09 |
| rs7759938 | 6 | 105378954 | C/T | 0.330 | 0.373 | 0.9 |
| rs7821178 | 8 | 78093837 | C/A | 0.676 | 0.658 | 1.0 |
| rs10453225 | 9 | 108920220 | G/T | 0.667 | 0.700 | 0.06 |
| rs2090409 | 9 | 108967088 | C/A | 0.667 | 0.688 | 0.08 |
| rs10739221 | 9 | 109060830 | C/T | 0.759 | 0.770 | 0.5 |
| rs12915845 | 15 | 89042467 | C/T | 0.582 | 0.582 | 0.3 |
| rs246185 | 16 | 14395432 | C/T | 0.323 | 0.300 | 0.6 |
| rs12446632 | 16 | 19935389 | A/G | 0.133 | 0.127 | 0.9 |

^a^Position based on GRCh37.p13 assembly.

^b^N = 21476.

**Table S6.** **Meta-analysis of the pubertal development genetic risk score (12 SNPs) and prostate cancer risk, stage and grade in the PRACTICAL consortium.**

| **trait** | **N** | **N_0_** | **N_1_** | **OR** | **95% CI** | **p-value** | **I^2^ (%)** | **p-value** |
| --- | --- | --- | --- | --- | --- | --- | --- | --- |
| control/case (0/1) | 36066 | 18536 | 17530 | 0.97 | (0.94,1.00) | 0.03 | 23.3 | 0.2 |
| Gleason score (0:≤6/1:≥7) | 16653 | 8310 | 8343 | 0.98 | (0.95,1.02) | 0.39 | 32.5 | 0.08 |
| stage (0:localized/1:advanced) | 17084 | 12843 | 4241 | 0.95 | (0.91,1.00) | 0.03 | 0.0 | 0.7 |

ORs indicate effects per tertile increase in genetic score adjusted for age and 15 principal components describing population structure.

**Figure S1.** **Histogram of pubertal development genetic risk scores in ProtecT (A), PRACTICAL (B).**

**Figure S2. Funnel plot of minor allele frequency-corrected genetic associations with Tanner stage against causal estimates for high grade prostate cancer obtained from each SNP in the pubertal development genetic risk score (ProtecT study).**

**
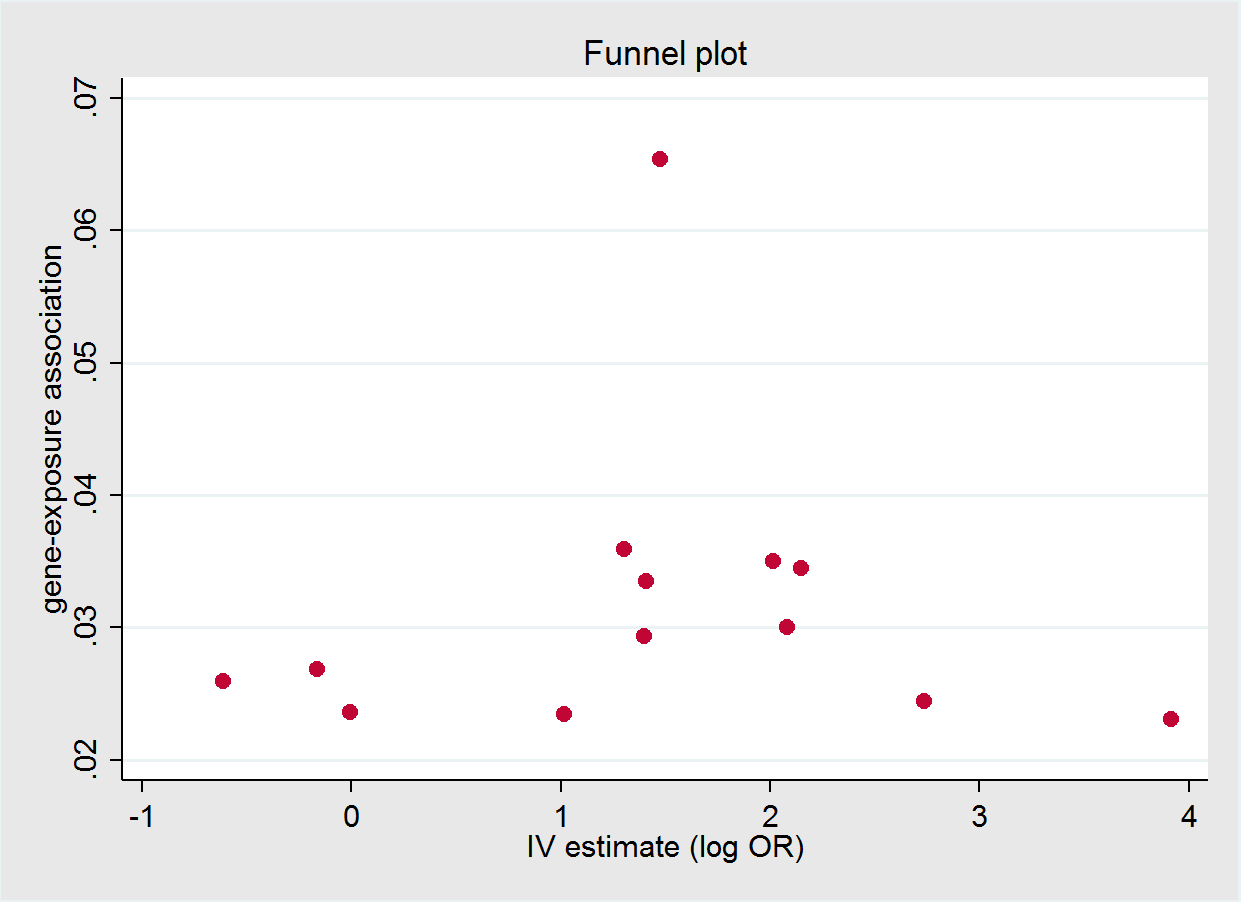
**

The pooled estimate for the causal effect of a unit increase in Tanner stage in ~13-15 year old boys on high grade prostate cancer corresponds to a log OR (± standard error) of 1.49 ± 0.47 (allele score with summarized data[1], p = 0.002) or 1.81 ± 1.26 (MR-Egger regression[2]; p = 0.18). The value of the intercept in the MR-Egger regression was -0.02 ± 0.09 (p = 0.79), thus suggesting that directional pleiotropy is unlikely to have biased the causal estimate.

**Figure S3. Kaplan-Meier 15-year survival estimates for prostate cancer-specific mortality and tertiles of the pubertal development genetic risk score, in the PRACTICAL consortium.**


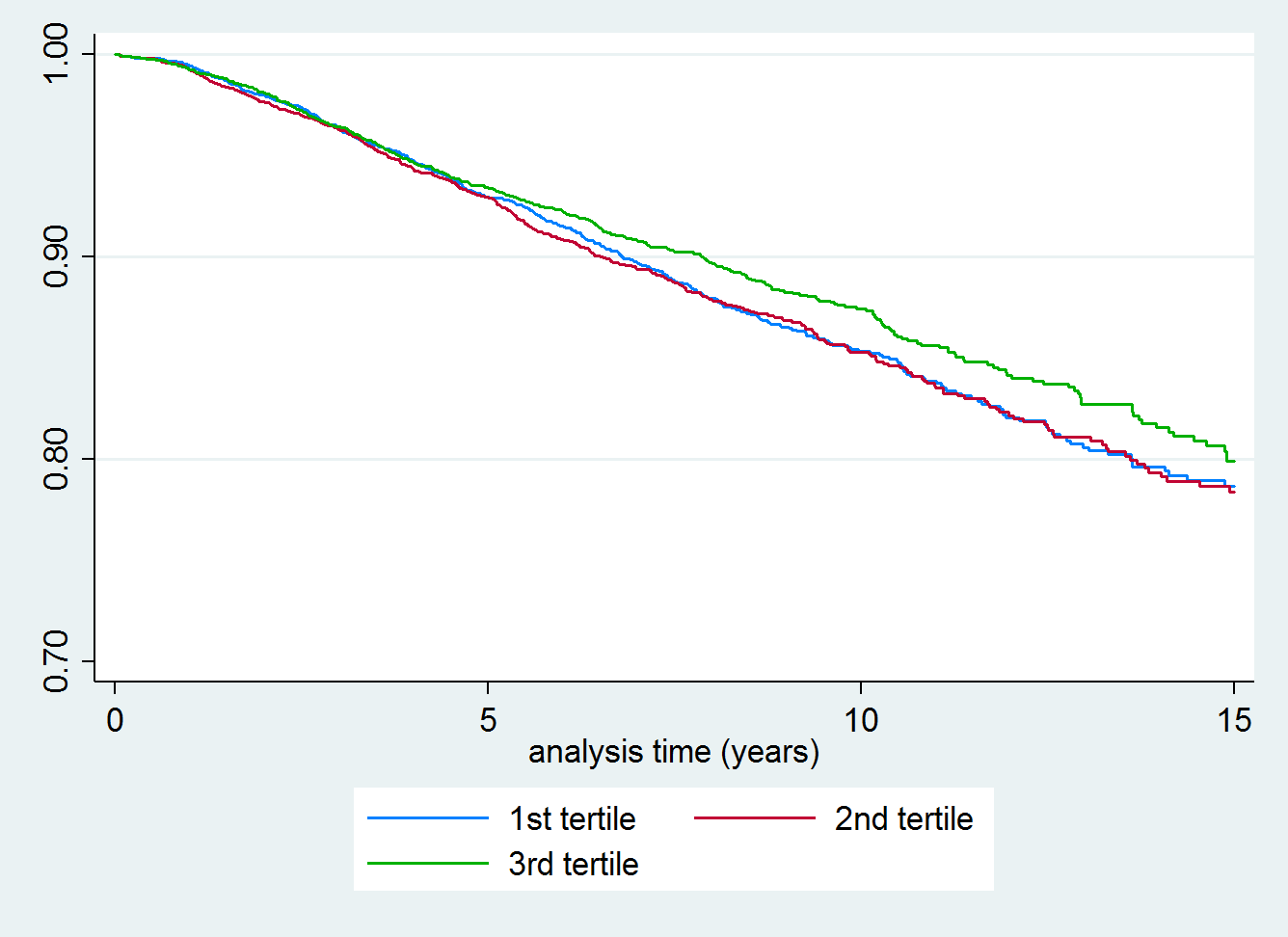


Model adjusted for age at diagnosis and 15 principal components.

Number of individuals = 13986

Number of failures = 1489

Years at risk = 99548

**Figure S4. Funnel plot of minor allele frequency-corrected genetic associations with Tanner stage against causal estimates for prostate cancer-specific mortality obtained from each SNP in the pubertal development genetic risk score (PRACTICAL consortium).**


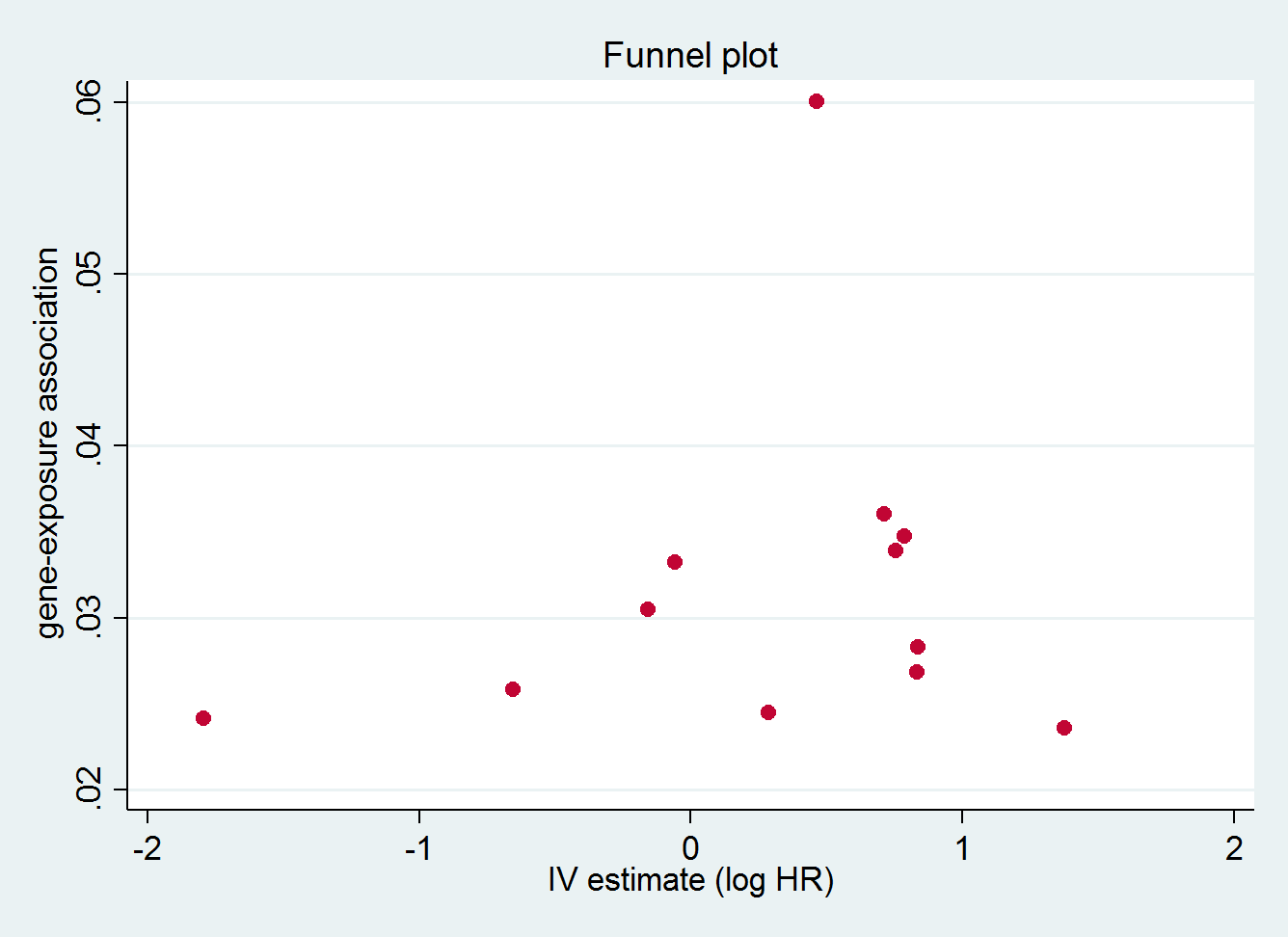


The pooled estimate for the causal effect of a unit increase in Tanner stage in ~13-15 year old boys on 15-year prostate cancer-specific mortality corresponds to a log OR (± standard error) of 0.48 ± 0.12 (allele score with summarized data[1], p < 0.001) or 0.88 ± 0.37 (MR-Egger regression[2], p = 0.04). The value of the intercept in the MR-Egger regression was -0.03 ± 0.03 (p = 0.3), thus suggesting that directional pleiotropy is unlikely to have biased the causal estimate. The slight asymmetry in the funnel plot is due to the effect of rs7821178, which is in the opposite direction to most of the other SNPs. Some heterogeneity between the causal estimates for the different genetic variants was evident (I^2^ = 49%; p = 0.03), but was almost halved when rs7821178 was excluded (I^2^ = 27%; p = 0.2).

**Supplementary Methods**

**ProtecT (Prostate Testing for cancer and Treatment)**

Genotyping and imputation

Tanner stage SNPs were obtained from genome-wide genotyping of ProtecT samples carried out on 3,390 individuals at the Center National de Génotypage, Evry, France, using the Illumina Human660W-Quad_v1_A array (Illumina, Inc., San Diego, CA) and subsequent genotype imputation.

The quality control process performed before imputation excluded individuals on the basis of the following: sex mismatches, minimal (< 0.325) or excessive heterozygosity (> 0.345), disproportionate levels of individual missingness (> 3%), cryptic relatedness measured as proportion of identity by descent (IBD > 0.1), and insufficient sample replication (IBD < 0.8). The remaining individuals were assessed for evidence of population stratification by multidimensional scaling analysis and compared with HapMap II (release 22) European descent (CEU), Han Chinese (CHB), Japanese (JPT) and Yoruba (YRI) reference populations; all individuals with non-European ancestry were removed. SNPs with a minor allele frequency (MAF) below 1%, a call rate of < 95% or evidence for violations of Hardy-Weinberg equilibrium (p < 5x10^-7^) were discarded.

Autosomal genotypic data were then imputed using Markov Chain Haplotyping software (MACH v.1.0.16[3]) and phased haplotype data from CEU individuals (HapMap release 22, Phase II NCBI B36, dbSNP 126) based on a cleaned dataset of 3,186 individuals and 514,432 autosomal SNPs. After imputation, all SNPs with indication of poor imputation quality (r^2^ hat < 0.30) were removed. X chromosome imputation was performed on a cleaned dataset of 3,186 individuals and 10,092 X chromosome SNPs, using MACH v.1.0.16 and MiniMac v.4.4.3, in conjunction with phased haplotype data from CEU individuals (HapMap 3 release 2, NCBI B36, dbSNP 126). The working dataset consisted of 2,927 individuals (1136 cases, 1791 controls) of European descent. Genotypic dosages, which represent the expected number of one of the alleles and range from 0 to 2, were derived from genome-wide data and used in the analysis. Dosages are continuous variables that incorporate the uncertainty of the imputation process.

Population stratification

The top 10 principal components that reflect the population’s genetic structure were estimated according to Price et al.[4] from genome-wide SNPs genotyped, imputed and cleaned as described above. All 10 principal components were included as covariates in the regression models to account for confounding by population stratification.

Covariables

Self-reported data on history of benign prostatic hyperplasia (BPH), having diabetes, family history of prostate cancer, birthweight, height and weight [to calculate body mass index (BMI) kg/m^2^], and inside leg length, were collected from questionnaires completed before receipt of the initial PSA test result. PSA was measured using standard methods in hospital laboratories at each of the study centres. Blood samples for IGF measurement were drawn at the time of the PSA test, frozen at -80^o^C within 36 hours, then transferred for assay on dry ice. Concentrations of IGF-I, IGF-II, and IGF binding protein (BP)-3 were measured by radioimmunoassay (RIA) which determines total IGF-I, IGF-II, or IGFBP-3 levels. Serum IGFBP-2 was measured using a one-step sandwich ELISA (DSL- 10-7100; Diagnostic Systems Laboratories)[5]. All assays were carried out blind to knowledge of case or control status. IGF-I:IGFBP-3 molar ratio was calculated as 0.13*[IGF-I]/0.36*[IGFBP-3].

Statistical analysis

The SNPs in the score were tested for deviation from Hardy-Weinberg equilibrium using the *hwsnp* function in the statistical package Stata, with a Bonferroni correction for multiple testing (p-value cut off = 0.004). Linkage disequilibrium between SNPs in the same gene was assessed with the SNAP program (<https://www.broadinstitute.org/mpg/snap/>) using data from the 1000 Genomes panel for the European HapMap CEU population,

We examined associations of the genetic score with traits which are either known prostate cancer risk factors or are associated with pubertal development: age, family history, PSA concentration, height, weight, BMI, birthweight, circulating IGF-I, IGF-II, IGFBP-2, and IGFBP-3 levels, IGF-I:IGFBP-3 molar ratio, BPH and diabetes. Associations between the puberty genetic score and the variables listed above were assessed using linear o logistic regression, adjusted for age, recruitment centre and 10 principal components. To be able to infer a causal effect of pubertal development on prostate cancer, the genetic score is expected not to be associated with potential confounders, unless they lie in the causal pathway to the disease. Additionally, we tested the association of the genetic score with leg length, as a marker of the timing of puberty[6], using a linear regression model as described.

Instrumental variable analysis

We estimated the causal effect of a unit decrease in Tanner stage (for the same age) on the odds of progression from low to high grade prostate cancer and 20-year prostate cancer-specific mortality using published data on the genetic association of each SNP with the exposure and their association with the outcome in ProtecT and PRACTICAL[1]. We reproduced the effect on Tanner stage of a weighted genetic score with summarized SNP data from the literature, taking into account linkage disequilibrium between some of the variants. This method has been shown to provide unbiased estimates with either equal or externally-derived weights and has the advantage of not requiring individual-level data[1].

We assessed the potential violation of the instrumental variable assumption of exclusion-restriction (i.e. no direct effect of the genetic variants on the outcome) using MR-Egger regression, which provides a valid test of directional –or unbalanced- pleiotropy and a consistent estimate of the true causal effect[2]. The intercept in MR-Egger regression, if different from zero, represents evidence of directional pleiotropy, whereas the slope corresponds to the true causal effect.

**Replication dataset**

**PRACTICAL consortium (PRostate cancer AssoCiation group To Investigate Cancer-Associated aLterations in the genome)**

Subjects

Fifteen studies in the consortium were from Europe, six from North America and two from Australia, and consisted of populations of mainly European ancestry[7]. ProtecT (and the allied ProMPT study) contributed to PRACTICAL, but were excluded from the replication analysis. There was a mixture of population- or hospital-based case-control studies, case-control studies nested within population-based cohorts, and cancer registry-based studies. Fourteen studies stated method of case ascertainment, and comprised 23% screen-detected and 77% clinically-detected cases. Data on cancer stage, grade and method of diagnosis was collected by each study independently. We categorized cancers as localized (T1 or T2 on TNM staging, or if not available, “localized” on SEER staging) or advanced (T3 or T4 on TNM staging, or if not available, “regional” or “distant” on SEER staging). The stage and grade of the cancers differed by study: of those reporting TNM stage (n=20), between 0% and 65% were advanced (N=4376); of those reporting grade (n=21), between 28% and 84% were grade 7 or more (N=8584).

Genotyping and imputation

Genotyping of PRACTICAL samples was carried out using an Illumina Custom Infinium genotyping array (iCOGS), designed for the Collaborative Oncological Gene-Environment Study (COGS) and consisting of 211,155 SNPs[7]. This array was designed to evaluate associations of genetic variants with breast, ovarian and prostate cancer (85,278 were specifically chosen for their potential relevance to prostate cancer). Participants with low call rates (< 95%) and high or low heterozygosity (p < 1×10^−5^) were excluded; a total of 201,598 SNPs passed quality control for the European ancestry samples. The details of the iCOGS array can be found on the COGS website (<http://www.cogseu.org>).

Imputation of ~17 million SNPs/indels using the 1000 Genomes Project (March 2012 release) as a reference panel was performed with the program IMPUTE v.2[8]. Polymorphisms with quality information scores of > 0.3 and MAF > 0.5% were taken forward for analysis[9].

We investigated the association of a puberty genetic score with prostate cancer risk and aggressiveness in a meta-analysis of men from 21 independent studies.

We calculated ORs for the association of the puberty genetic score with prostate cancer incidence, grade (Gleason score ≥ 7 vs ≤ 6) and stage (advanced vs localized) using logistic regression, and hazards ratios (HR) for survival using Cox proportional hazards regression. All regression models were run including only individuals of white ethnicity and were adjusted for age and the top 15 principal components describing population stratification. Results from each study were then meta-analyzed using the Stata *metan* command assuming a fixed-effects model, with the exception of the survival analysis for which there were low sample sizes in the majority of studies and therefore no stratification by study was carried out.

**Definitions**

**Directional pleiotropy:** it takes place when the pleiotropic effects of the genetic variants used as instruments in Mendelian randomization analyses are not balanced about the null[2].

**MR-Egger regression and test**: equivalent of Egger regression and test for small study bias in meta-analysis used in Mendelian randomization to assess whether the causal estimates obtained with genetic variants that are weak instruments are more skewed than those obtained from stronger genetic variants. The intercept in MR-Egger regression represents an estimate of the average pleiotropic effect across genetic variants and, if different from zero, provides evidence of the existence of overall directional pleiotropy[2].

**Supplementary References**

1. Burgess S, Scott RA, Timpson NJ, Davey Smith G, Thompson SG: **Using published data in Mendelian randomization: a blueprint for efficient identification of causal risk factors**. *Eur J Epidemiol* 2015, **30**:543–552.

2. Bowden J, Davey Smith G, Burgess S: **Mendelian randomization with invalid instruments: effect estimation and bias detection through Egger regression**. *Int J Epidemiol* 2015, **44**:512–525.

3. Li Y, Willer CJ, Ding J, Scheet P, Abecasis GR: **MaCH: using sequence and genotype data to estimate haplotypes and unobserved genotypes.** *Genet Epidemiol* 2010, **34**:816–34.

4. Price AL, Patterson NJ, Plenge RM, Weinblatt ME, Shadick NA, Reich D: **Principal components analysis corrects for stratification in genome-wide association studies.** *Nat Genet* 2006, **38**:904–9.

5. Rowlands M-A, Holly JMP, Gunnell D, Donovan J, Lane JA, Hamdy F, Neal DE, Oliver S, Davey Smith G, Martin RM: **Circulating insulin-like growth factors and IGF-binding proteins in PSA-detected prostate cancer: the large case-control study ProtecT.** *Cancer Res* 2012, **72**:503–15.

6. Sandhu J, Ben-Shlomo Y, Cole TJ, Holly J, Davey Smith G: **The impact of childhood body mass index on timing of puberty, adult stature and obesity: a follow-up study based on adolescent anthropometry recorded at Christ’s Hospital (1936-1964).** *Int J Obes* 2006, **30**:14–22.

7. Kote-Jarai Z, Easton DF, Stanford JL, Ostrander EA, Schleutker J, Ingles SA, Schaid D, Thibodeau S, Dörk T, Neal D, Cox A, Maier C, Vogel W, Guy M, Muir K, Lophatananon A, Kedda MA, Spurdle A, Steginga S, John EM, Giles G, Hopper J, Chappuis PO, Hutter P, Foulkes WD, Hamel N, Salinas CA, Koopmeiners JS, Karyadi DM, Johanneson B, et al.: **Multiple novel prostate cancer predisposition loci confirmed by an international study: the PRACTICAL consortium**. *Cancer Epidemiol Biomarkers Prev* 2008, **17**:2052–2061.

8. Howie BN, Donnelly P, Marchini J: **A flexible and accurate genotype imputation method for the next generation of genome-wide association studies.** *PLoS Genet* 2009, **5**:e1000529.

9. Al Olama AA, Kote-Jarai Z, Berndt SI, Conti D V, Schumacher F, Han Y, Benlloch S, Hazelett DJ, Wang Z, Saunders E, Leongamornlert D, Lindstrom S, Jugurnauth-Little S, Dadaev T, Tymrakiewicz M, Stram DO, Rand K, Wan P, Stram A, Sheng X, Pooler LC, Park K, Xia L, Tyrer J, Kolonel LN, Le Marchand L, Hoover RN, Machiela MJ, Yeager M, Burdette L, et al.: **A meta-analysis of 87,040 individuals identifies 23 new susceptibility loci for prostate cancer**. *Nat Genet* 2014, **46**:1103–1109.
